# Accommodating population stratification in case-control association analysis: a new test and its application to genome-wide study on rheumatoid arthritis

**DOI:** 10.1186/1753-6561-3-s7-s111

**Published:** 2009-12-15

**Authors:** Yufang Zhang, Xiangjun Xiao, Kai Wang

**Affiliations:** 1 Program of Public Health Genetics, University of Iowa, 200 Hawkins Drive E177 GH, Iowa City, Iowa 52242 USA; 2 Department of Biostatistics, University of Iowa, 200 Hawkins Drive C227 GH, Iowa City, Iowa 52242 USA

## Abstract

It is well known that conventional association tests can lead to excessive false positives when there is population stratification. We propose a new test for detecting genetic association with a case-control study design. Unlike some other methods for handling population stratification, we treat the cases as a population and the controls as another one even though each of them may be a mixture of several sub-populations. A likelihood-ratio test is used to test whether the allele frequency of a testing single-nucleotide polymorphism in the case population is the same as that in the control population. This new test is applied to the Genetic Analysis Workshop 16 Problem 1 data on rheumatoid arthritis. Compared with the Pearson chi-square genotype test, the association strength of many single-nucleotide polymorphisms is decreased while the signal at the HLA region on 6p21 is maintained.

## Background

One well known drawback of case-control study design in genetic association studies is that it may be affected by population stratification. Population stratification is an ethnic confounder. If a sample population is from a recent mixture of different ethnic subpopulations, it may make the cases and controls have different genetic background and spurious association may occur. In order to control the effect of population stratification, genome control [1], structured association [2], and principal components [3] are usually used. These methods try to gather information on population structure from markers not associated with the phenotype (null markers). In this paper, we introduce a likelihood-ratio test for genetic association in the presence of population stratification. This method does not make assumptions on the number of sub-populations in cases or in controls, nor does it make use of null markers. This method is then applied to the Genetic Analysis Workshop 16 (GAW16) Problem 1 data set.

## Methods

Let FST denote the correlation of alleles drawn from a common subpopulation [4]. FST is the proportion of the total heterozygosity in the population due to the differences in allele frequencies among each subpopulation. It can be expressed as FST = , where  is the average allele frequency over all subpopulations and *V*(*p*) is the variance of allele frequency *p *among subpopulations. The genotype frequencies in a population are jointly determined by FST and the frequency of a reference allele, say *A*. We treat cases as samples from one population and controls as samples from another. Let *F*_1 _be the value of FST in cases and *F*_2 _be the value of FST in controls. The *A *allele frequency in cases and in controls are denoted by *p*_1 _and *p*_2_, respectively. Let *a *denotes the other allele, the frequencies of genotypes *AA*, *Aa*, and *aa *in cases and controls are presented in Table 1.

**Table 1:** Genotype frequencies in cases and controls

	*AA*	*Aa*	*aa*
Cases	*p*_12 _= *F*_1_*p*_1_+(1-*F*_1_)*p*_1_^2^	*p*_11 _= 2(1-*F*_1_)*p*_1_(1-*p*_1_)	*p*_10_* = F*_1_*(*1-*p*_1_)+(1-*F*_1_)(1-*p*_1_)^2^
Controls	*P*_22 _= *F*_2_*p*_2_+(1-*F*_2_)*p*_2_^2^	*p*_21 _= 2(1-*F*_2_)*p*_2_(1-*p*_2_)	*p*_20_* = F*_2_(1-*p*_2_)+(1-*F*_2_)(1-*p*_2_)^2^

We proposed a likelihood ratio test to test the hypotheses H0: *p*_1 _= *p*_2 _= *p*, *F*_1_, *F*_2 _versus H_*A*_: *p*_1 _≠ *p*_2_, *F*_1_, *F*_2_. *F*_1 _and *F*_2 _are treated as nuisance parameters. The log-likelihood function is

in which *i *= 1 or 2 for cases or controls; for each marker genotype, *j *= 0, 1, or 2 for zero *A *allele, one *A *allele, or two *A *alleles, respectively. *n*_*ij *_are observed genotype counts and *p*_*ij *_are genotype frequencies as listed in Table 1.

The maximization of the likelihood function *L*(*p*_1_, *p*_2_, *F*_1_, *F*_2_) under the alternative hypothesis is straightforward. The maximized estimate of each genotype frequency happens to be the observed genotype frequency in cases and controls. However, there is no explicit solution to the maximization problem under the null hypothesis. To maximize the log-likelihood function under H_0_, we take the first-order partial derivatives of the log-likelihood function under the null with respect to *F1 *and *F2 *and set them to zero. Each of the two equations gives an expression of *F1 *or *F2 *in terms of *p*. Then a grid search (step size 0.001) over *p *ranging from 0.001 to 0.999 is used to find the best value of *p *maximizing the null log-likelihood function.

The likelihood ratio test statistic is

According to standard statistical theory, it asymptotically follows a chi-square distribution with 1 degree of freedom.

## Results

**Figure 1:**
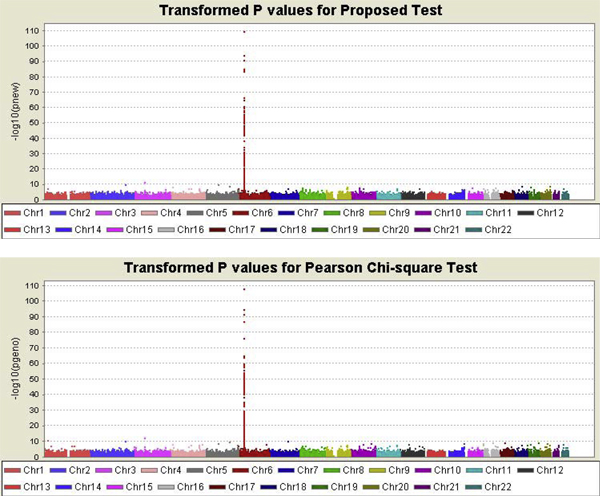
**Plots of transformed *p*-values for the new test and Pearson chi-square test**.

**Figure 2:**
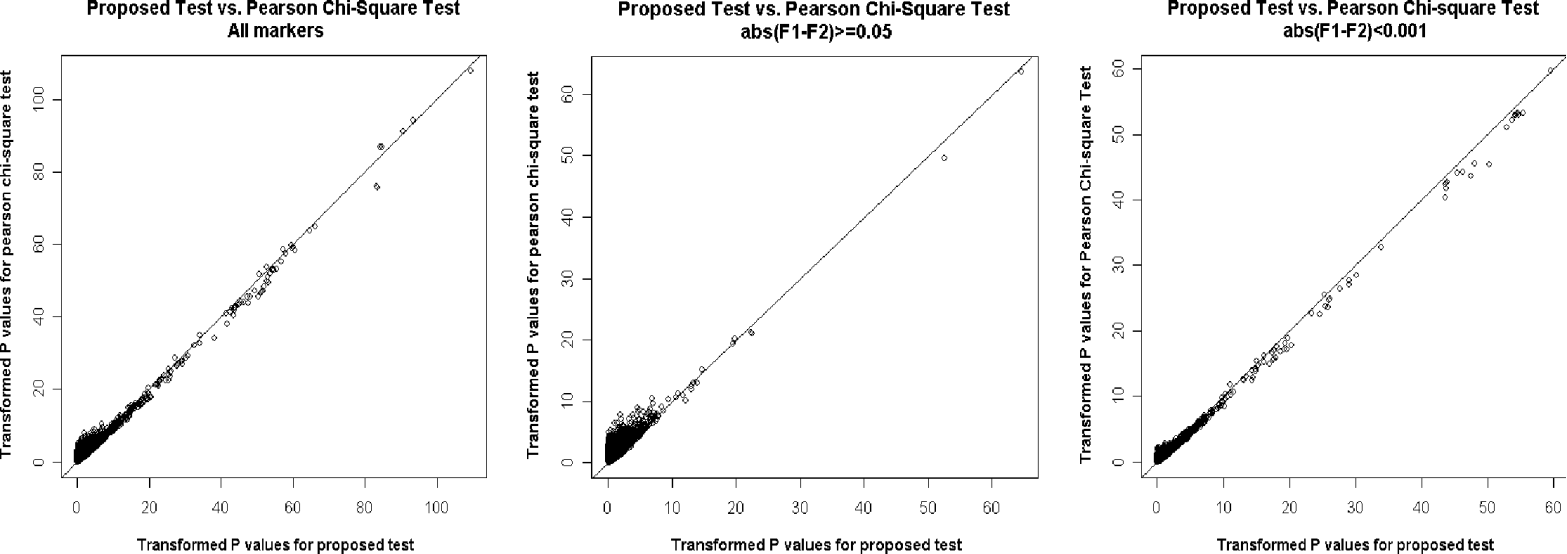
**Comparison of transformed *p*-values between new test and chi-square test**.

The GAW16 Problem 1 data set consists of 545,080 SNP markers throughout the genome for 2062 unrelated individuals consisting of 868 patients with rheumatoid arthritis and 1194 controls. After quality control, 65,372 (11.99%) markers were removed. A marker was removed if it met any one of the following criteria: its call rate was less than 90%; the minor allele frequency was less than 0.01, or it did not follow Hardy-Weinberg equilibrium in controls (at significance level 0.05). In addition, we only considered markers on autosomal chromosomes. Finally, 466,317 markers were included for further study. Transformed *p*-values (-log_10_(*p*)) were plotted genome-wide in Figure 1 (top panel) by using the *Haploview *program. In addition, for comparison, results from traditional Pearson chi-square test were also plotted (Figure 1, bottom panel). Figure 2 plots the transformed *p*-values for the proposed test versus that for the Pearson chi-square test. The left-most panel includes all markers. The middle panel includes only those markers for which the absolute value of the difference in the estimated *F*_1 _and *F*_2 _is larger than or equal to 0.05. The right-most panel includes markers with difference between *F*_1 _and *F*_2 _less than 0.001. If the difference is within 0.001, we treat *F*_1 _and *F*_2 _are approximately equal. When the difference between *F*_1 _and *F*_2 _becomes larger, the proposed test statistic tends to be less significant.

## Discussion

We proposed a test for genetic association study in the presence of population stratification. Population stratification is a confounder to the difference of genotype frequencies between cases and controls. Unlike some other methods such as the structured association, the proposed test does not try to classify each individual. Instead, it allows for the difference in the composition of cases and controls by using two of FST coefficients, one for cases and one for controls. Population genetics suggests that the FST for a natural population may be small (for instance, 0.001 or 0.01). This may be true for controls, but no longer true for a selected sample such as cases. It is easy to construct a case sample for which the FST is 0.8 or higher. Our test provides a simple way to reduce the confounding impact of population stratification compared with the Pearson chi-square statistic.

The proposed method attributes any deficiency in heterozygosity in cases or controls to population stratification. Its power to detect association can be compromised when there is no population stratification, especially when the trait is recessive [5]. Because population stratification affects not only FST but also allele frequencies in cases and controls, the proposed method cannot completely eliminate the confounding effect of population stratification. Due to the page limitation, no simulation results comparing the proposed method and the Pearson's chi-square statistic are reported. One reviewer pointed out that this may make it difficult to interpret the difference between these two methods observed in current study. In our unreported simulation studies, the proposed method is still more robust to population stratification than Pearson's chi-square statistic.

## Conclusion

A method for detecting association in the presence of population stratification is proposed. Analysis of the GAW16 Problem 1 data on rheumatoid arthritis suggests it is more robust to population stratification than the Pearson's chi-square statistic. The proposed test is implemented in two computer languages, *C++ *and *R*. Both versions are available from the authors upon request.

## List of abbreviations used

GAW16: Genetic Analysis Workshop 16.

## Competing interests

The authors declare that they have no competing interests.

## Authors' contributions

YZ participated in the design of the study, implemented the test, and drafted the manuscript. XX participated in the data collection and design of the study. KW conceived of the study, participated in the design of the study, helped to draft the manuscript, and advised in the process of the study.
